# The Impact of MSTN Gene Editing on Meat Quality and Metabolomics: A Comparative Study Among Three Breeds of MSTN-Edited and Non-Edited Cattle

**DOI:** 10.3390/ani15010047

**Published:** 2024-12-27

**Authors:** Shan Luo, Yuanqing Liu, Lige Bu, Dezheng Wang, Zhaoyu Wen, Yuqing Yang, Yanan Xu, Di Wu, Guangpeng Li, Lei Yang

**Affiliations:** State Key Laboratory of Reproductive Regulation and Breeding of Grassland Livestock, College of Life Science, Inner Mongolia University, Hohhot 010021, China; luoshan0112@126.com (S.L.); 22408011@mail.imu.edu.cn (Y.L.); ligebu1996@163.com (L.B.); 18906485675@163.com (D.W.); wenzhaoyuimu@163.com (Z.W.); yyq284219588@163.com (Y.Y.); 17824230613@163.com (Y.X.); wudi2020imu@163.com (D.W.)

**Keywords:** MSTN, gene editing, meat quality traits, metabolomics

## Abstract

Myostatin (MSTN) serves as a negative regulatory factor for muscle development. A reduction in MSTN gene expression can enhance muscle mass and increase meat production. However, whether it will impact meat quality traits remains one of the major concerns in the cattle breeding industry. To explore the meat quality traits of MSTN gene-edited cattle, this study compared the meat quality traits of three MSTN gene-edited cattle breeds with those of non-gene-edited cattle, including Luxi, Angus, and Mongolian cattle, and further conducted an analysis in combination with metabolomics. We found that MSTN gene-edited cattle were superior to non-gene-edited cattle in terms of meat pH, shear force, and intramuscular fat content. However, no significant differences were observed in water-holding capacity, water content, and protein content of the meat. Metabolomics analysis revealed three upregulated differential metabolites common to MSTN gene-edited and non-gene-edited cattle across three breeds, namely lactoyl-valine, 3-phenylactic acid, and lactoyl-methionine. Some studies have indicated that these metabolites can improve the meat’s flavor. In this study, we compared the meat quality traits and metabolomics data between MSTN gene-edited and non-gene-edited cattle, and found that the meat quality of MSTN gene-edited cattle was superior to that of non-gene-edited cattle.

## Simple Summary

A reduction in MSTN gene expression can promote muscle growth, but whether it affects meat quality traits while increasing meat yield is one of the main concerns in the beef cattle breeding industry. Therefore, this study investigates the meat quality traits and metabolomics of MSTN gene-edited cattle compared to non-gene-edited cattle across three breeds. The aim is to explore the impact of MSTN gene editing on meat quality in these three cattle breeds, providing new insights into the meat quality of gene-edited beef.

## 1. Introduction

Luxi cattle is one of the five major local yellow cattle breeds in China (Qinchuan cattle, Nanyang cattle, Luxi cattle, Yanbian cattle, and Jinnan cattle); it has the characteristics of strong coarse feed utilization, good fattening performance, tender meat, and obvious marbling [1,2]. Angus cattle are indigenous to Scotland in the UK and are known for their delicate yet firm muscles with an even marbling distribution that results in a mellow and juicy taste [3]. Mongolian cattle have various uses in the north of China, such as agricultural activities, meat production, and milk production due to their cold resistance, disease tolerance, rough feeding resistance, and strong adaptability [4]. However, Luxi cattle have the disadvantages of slow growth, poor hindquarters development, and low meat production [5]; Angus cattle have high intramuscular fat content; and Mongolian cattle exhibit underdeveloped hindquarters, low meat production, and slow weight gain [6]. The MSTN gene serves as a negative regulator of muscle growth and development [7], and its mutation can induce the rapid development of skeletal muscle and the formation of double muscle phenotypes. Using gene editing technology to target mutations in the MSTN gene can significantly increase meat production in meat livestock such as cattle and pigs, while reducing intramuscular fat content [8,9], effectively addressing the problems of breeding. However, whether meat quality will be affected when meat production is improved remains a primary concern of the cattle breeding industry.

In the evaluation of meat quality, the commonly used indexes include pH value, tenderness, water-holding capacity, nutrient content, and so on. The pH value of muscle is generally regarded as a criterion for evaluating the quality of muscle and is one of the significant indicators and key parameters for assessing meat quality. The pH value can influence parameters such as meat color, shear force, tenderness, and tissue state of the muscle. Tenderness is one of the key indicators of meat quality for food use, and is one of the main factors affecting consumer satisfaction and acceptance [10]. The degree of tenderness can be indicated by the magnitude of shear force, with smaller shear force indicating higher tenderness. The water-holding capacity is the ability of the muscle to maintain its original water and added moisture when subjected to external forces, such as pressurization, chopping, heating, salting, and other processing or storage conditions. A large number of previous studies have shown that the size of the hydraulic force is related to factors such as muscle maturation time, protein degradation, pH value, etc. [11,12,13]. In the industry standards of the Ministry of Agriculture of China, there are four main methods for the determination of tethering force: water loss by the pressure method, water loss by the centrifugal method, drip loss, and cooking loss. In this study, water loss by pressure method and cooking loss were used to determine the water-holding capacity of the muscle. The protein content in meat is related to its nutritional value and plays an important role in the body’s metabolic process and growth and development. The fat content in muscle has a certain correlation with the palatability, flavor, tenderness, and juiciness of meat. Beef contains about 20% protein and the fat content is usually between 5 and 30%, making it a nutrient-rich high-protein food.

The meat quality trait is a complex and comprehensive index, which is affected by many factors. Genomics can deeply analyze the regulation mechanism of meat quality traits and provide new ideas and methods for meat quality improvement. Metabolomics is the study of metabolites in tissues and changes in metabolites due to different factors [14]. Changes in metabolite species and content can form a more direct relationship with changes in animal phenotype, and metabolomics is the closest to phenotypic changes [15]. In animal husbandry, using metabolomics technology to quantitatively and qualitatively analyze the small molecule metabolites of livestock and poultry can provide a comprehensive and in-depth understanding of the physiological mechanisms of economically important traits in livestock and poultry [16]. Non-targeted metabolomics enables comprehensive analysis of endogenous small molecule metabolites in organisms [17]. And data analysis is usually performed using multivariate statistical analysis methods such as principal component analysis (PCA) and partial least squares discriminant analysis (PLS-DA), and is combined with the *t*-test *p*-value and variable projection importance value (VIP) to screen for differential metabolites or potential molecular markers. The screened metabolites are then utilized for clustering analysis, metabolic pathway enrichment analysis, and so on [18]. Currently, metabolomics technologies have been widely used in the study of the economic status of livestock and poultry; however, studies combining metabolomics analysis of meat quality traits in MSTN gene-edited cattle have rarely been reported. Therefore, in this study, MSTN gene-edited cattle and non-gene-edited cattle were selected for meat quality traits, and non-targeted metabolomics technology was employed to conduct comprehensive analysis, identification, and comparison of their metabolites, screening out differential metabolites and metabolic pathways, uncovering the metabolomics characteristics of meat quality traits of MSTN gene-edited cattle and non-gene-edited cattle, and providing new data for the meat quality of MSTN gene-edited cattle.

## 2. Materials and Methods

### 2.1. Experimental Animals

Our team used CRISPR/Cas9 technology and somatic cell nuclear transfer technology to produce MSTN gene-edited Luxi cattle, Angus cattle, and Mongolian cattle. A total of 36 two-year-old bulls were used in this study. Six MSTN gene-edited Luxi cattle (L-MT), six MSTN gene-edited Angus cattle (A-MT), and six MSTN gene-edited Mongolian cattle (M-MT) were selected as experimental groups, while six non-gene-edited Luxi cattle (L-WT), six non-gene-edited Angus cattle (A-WT), and six non-gene-edited Mongolian cattle (M-WT) were selected as control groups. All experimental animals were raised under the same feeding conditions at the Inner Mongolia University Germplasm Innovation and Breeding Base, from which we randomly selected subjects. Animal care and experimental protocols were approved by the Animal Ethics Committee of Inner Mongolia University (IMU-cattle-2023-065, 25 March 2023) and were carried out in accordance with the committee’s guidelines for animal research.

### 2.2. Sample Collection and Processing

After slaughter, about 1000 g of muscle of upper brain, eye meat, external spine, internal spine, anterior tendon, posterior tendon, and semitendinosus of each cattle were collected and transported back to the laboratory. They were subjected to 24 h acidification at 4 °C before the measurement of meat quality traits. Muscle samples of approximately 100 mg from the Longissimus thoracis of 36 cattle were collected, placed in a cryotube, and then frozen in liquid nitrogen for storage, used for non-targeted metabolomics analysis. All the collected samples were fed under the same conditions and underwent the same sample collection protocol.

### 2.3. Experimental Methods

#### 2.3.1. Meat Traits Measurement

The upper brain, eye meat, external spine, internal spine, anterior tendon, posterior tendon, and semitendinosus muscle tissues were taken. The pH values of different muscle parts were determined using a portable pH meter (PH5, Beijing Tianxiang Feiyu Technology Co., Ltd., Beijing, China) after 24 h acid removal at 4 °C. After sampling the muscle tissues of different parts along the muscle fiber direction using a sampler, the shear force of the muscle samples was measured using a muscle tenderness meter (C-LM3B, Beijing Tianxiang Feiyu Technology Co., Ltd., Beijing, China). After measuring the initial weight, pressure was applied perpendicularly to the muscle fiber direction using a carcass meat water-binding rate analyzer (Teno-voMeat-1, Beijing Tianxiang Feiyu Technology Co., Ltd., Beijing, China). After squeezing, the surface water was wiped dry with paper and weighed. The muscle squeezing loss rate was calculated based on the weight difference before and after. The weighed muscles of each part were placed in a cooking bag, put into a 75 °C water bath (GD120, Grant, UK) for 45 min, heating until the center of the meat sample temperature reached 70 ± 2 °C. Then, they were removed and cooled to room temperature, the meat was taken out of the bag, the surface was dried and weighed, and the rate of muscle cooking loss was calculated based on the difference between the before and after mass. About 100 g of muscle samples from each part were taken, homogenized into minced meat using a homogenizer, and then placed in a near-infrared meat quality analyzer (SERIES 3000, Next Instruments, Austin, TX, USA) to determine the water, protein, and intramuscular fat contents of each part of the muscle samples.

#### 2.3.2. Metabolite Extraction

The collected samples were thawed on ice and metabolites were extracted using 50% methanol buffer solution. Then 100 mg of sample was extracted with 1 mL of pre-cooled 50% methanol, vortexed for 1 min, and incubated at room temperature for 10 min; the extract was stored overnight at −20 °C. After centrifuging at 4000× *g* for 20 min, the supernatant was transferred to a new 96-well plate. The samples were stored at −80 °C before LC-MS analysis. In addition, 10 μL of each extraction solution was prepared as a mixed QC sample.

#### 2.3.3. LC-MS Analysis

The liquid chromatography system used for data was VanquishFlexUPLC, an ultra-high-pressure liquid-phase system from Thermo (Waltham, MA, USA), and the chromatographic column used was ACQUITYUPLCT3. During the analysis, the column temperature was set to 40 °C, and the flow rate was 0.3 mL/min. The mobile phase used was phase A: 5 mmol/L ammonium acetate + 5 mmol/L acetic acid + water; phase B: acetonitrile (LC-MS acetonitrile). The high-resolution tandem mass spectrometer used was Q-Exactive (Thermo Fisher Scientific, Bremen, Germany). Metabolites eluted from the column were detected. The shielding gas pressure of the ion source was 0 PSI, the gas 1 (auxiliary gas) pressure was 10 PSI and the gas 2 (sheath gas) pressure was 35 PSI, and the source temperature was 350 °C. For positive ion mode, the ion spray floating voltage was set to 4000 V; for negative ion mode, the ion spray floating voltage was set to −4500 V. The mode of data acquisition was DDA (information-dependent acquisition) mode. In one acquisition cycle, the primary acquisition range was 70–1050 Da, the primary resolution was 70 K (@m/z 200), the AGC target was 3 × 10^6^, and the Maximum IT was 100 ms. The top 3 signal ions with a signal accumulation intensity exceeding 100,000 from the primary spectra were selected for secondary fragmentation scanning. The secondary resolution was 17.5 K (@m/z 200), the Maximum IT was 50 ms, and the dynamic exclusion was set at 6 s. During the acquisition process, QC samples were scanned every 10 samples, and the quality gap between QCs was used to correct the systematic errors of a whole batch of experiments.

#### 2.3.4. Real-Time Fluorescence Quantitative PCR

Total RNA from the longest muscle tissue of the cow’s back was extracted using the RNA extraction kit (TIANGEN, Beijing, China) as a template. The mRNA was reverse transcribed into cDNA using the PrimeSptTM II 1st Strand cDNA Synthesis Kit (TaKaRa, Kyoto, Japan). According to the principles of quantitative primer design, quantitative primers for genes related to metabolic pathways (Table 1) were synthesized and used for qPCR determination. The primers were synthesized by Shenggong. The qPCR reaction system (20 μL): SYBR 10 μL, cDNA 1 μL, 10 μmol/L upstream and downstream primers 0.8 μL, and RNase Free ddH2O to complete the system. The qPCR reaction program: 95 °C 30 s (1 cycle), 95 °C 10 s, 60 °C 30 s (40 cycles), 95 °C 15 s, 60 °C 1 min, 95 °C 30 s, 50 °C 30 s (1 cycle). GAPDH was used as an internal reference gene, and the relative expression values of each gene mRNA were calculated using the 2^−ΔΔCt^ algorithm. This experiment was repeated 3 times.

#### 2.3.5. Data Statistical Analysis

The data on meat quality traits were statistically analyzed using SPSS 27, and the data were presented as mean ± standard. For metabolomics data, the XCMS software (version 4.4.0) was employed to conduct preprocessing of the collected mass spectrometry data, including peak picking, peak grouping, retention time correction, secondary peak grouping, isotope and adduct annotation, etc. The original LC-MS data files were converted to mzXML format and then processed using the XCMS, CAMERA, and metaX toolboxes implemented in R software (version 4.0). The statistical analyses were primarily accomplished using R software (version 4.0). The raw intensity values of metabolites would be normalized by median. The clustering heatmap was plotted by the R package pHeatmap (version 1.0.12). PCA analysis and significant differential metabolite analysis were carried out using the R package metaX (version 1.4.2). PLS-DA analysis was conducted by the R package ropls (version 1.38.0), and the VIP values of each variable were calculated. Correlation analysis was performed using the Pearson correlation coefficient in the R package cor (version 4.0). Differential pathway enrichment analysis based on the hypergeometric test was conducted for KEGG Pathway. *p* < 0.05 was the functional entry for significant enrichment of differential metabolites.

## 3. Results

### 3.1. Meat Quality Traits

To explore the meat quality traits of MSTN gene-edited cattle, we examined the pH, shear force, pressing loss rate, cooking loss rate, and nutritional components of the meat. A one-way analysis was conducted to compare the meat quality traits between MSTN gene-edited cattle and non-gene-edited cattle, and the results are shown in Table 2. Table 2 shows that there were significant differences (*p* < 0.05) between L-MT and L-WT, A-MT and A-WT, and M-MT and M-WT in terms of meat pH, shear force, and intramuscular fat content in different muscles. However, no significant differences were found in the pressing loss rate, cooking loss rate, water content, and protein content. The results indicate that compared with non-gene-edited cattle, the meat pH, shear force, and intramuscular fat content of the three types of MSTN gene-edited cattle were significantly reduced (*p* < 0.05).

### 3.2. Metabolomics

#### 3.2.1. Multivariate Statistical Analysis

**Figure 1:**
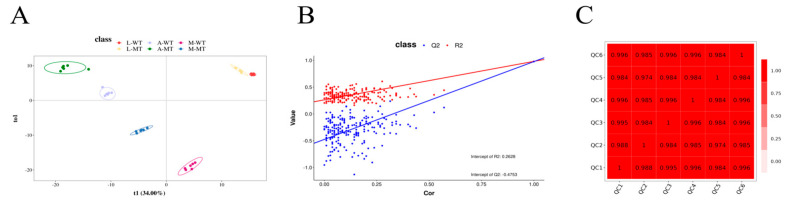
Multivariate statistical analysis. (**A**) PLS-DA score plot of L-MT vs. L-WT, A-MT vs. A-WT, and M-MT vs. M-WT; (**B**) results of the permutation test; (**C**) result of the quality control analysis.

**Figure 2:**
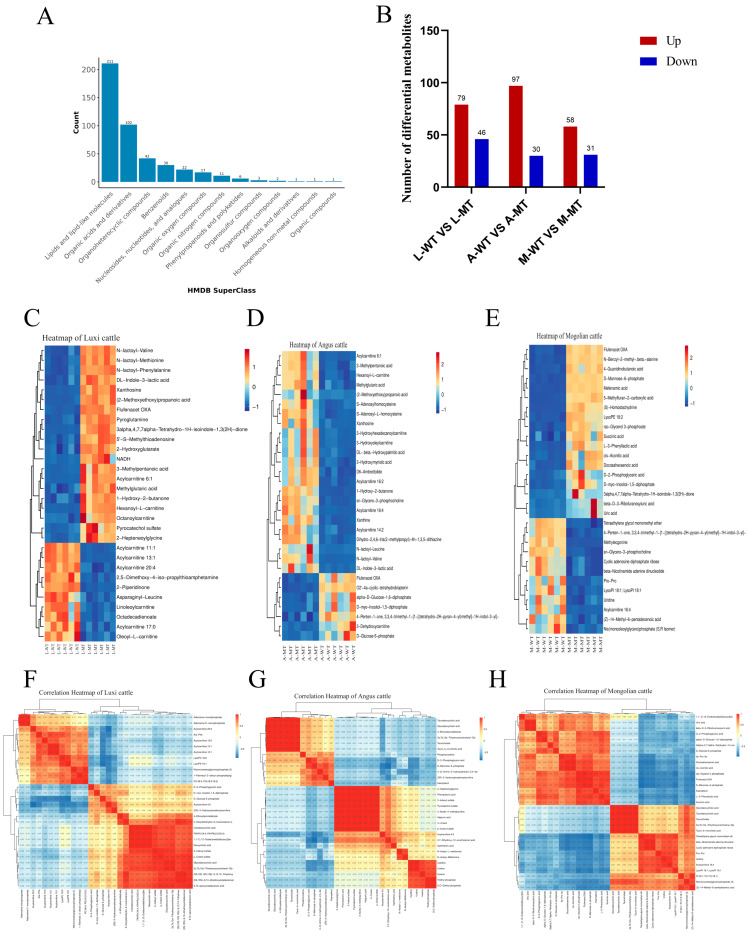
Analysis of differential metabolites. (**A**) statistical chart of HMDBSuperClass classification annotation of metabolites; (**B**) statistical chart of the identified significant differential metabolites. (**C**–**E**) heatmaps of differential metabolites between L-MT and L-WT, A-MT and A-WT, and M-WT and M-MT; (**F**–**H**) correlation heatmaps of differential metabolites between L-MT and L-WT, A-MT and A-WT, and M-WT and M-MT. In the heatmaps, red indicates positive correlation, and blue indicates negative correlation. The deeper the color, the stronger the correlation.

To determine the differences among sample groups, PLS-DA analysis was conducted. The results of the PLS-DA showed that samples within different groups tended to cluster, indicating good reproducibility, while clear separation between the groups suggested significant differences. Samples within different groups tended to cluster, and significant differences were observed between groups (*p* < 0.05), indicating that there was significant variability between MSTN gene-edited and non-gene-edited cattle. The permutation test plot (Figure 1B) indicted that the model did not have overfitting and had good stability. The QC samples showed good reproducibility (Figure 2C), and the instrument had good stability and high data quality throughout the detection process.

#### 3.2.2. Identification of Differential Metabolites

To classify the identified differential metabolites, classification annotations were carried out using HMDB (Figure 2A). A total of 211 metabolites were identified in lipids and lipid-like molecules, 102 metabolites in organic acids and derivatives, 42 metabolites in organic heterocyclic compounds, 30 metabolites in benzene compounds, and 30 metabolites in nucleosides, nucleotides, and analogues, and so on.

#### 3.2.3. Screening Results and Analysis of Significantly Differential Metabolites

Select metabolites with *p* < 0.05 and FC > 1.50 as the final significantly differential metabolites between L-MT and L-WT, A-MT and A-WT, and M-MT and M-WT (Figure 2B). Respectively, 125, 127, and 89 significantly differential metabolites were obtained. Compared with L-WT, 79 differential metabolites were upregulated and 46 were downregulated in L-MT. Compared with A-WT, 97 differential metabolites were upregulated and 30 were downregulated in A-MT. Compared with M-WT, 58 metabolites were upregulated and 31 were downregulated in M-MT. To explore the expression levels of differential metabolites between L-MT and L-WT, A-MT and A-WT, and M-MT and M-WT, hierarchical clustering analysis was performed for the top 30 differential metabolites (Figure 2C–E and Tables S1–S3). The results showed that 2-(4-fluoro-N-propyl-2-phenylphenylamino)-2-oxoacetic acid, lactoyl-methionine, etc., were highly expressed in L-MT, while octadecadienoic acid, oleyl L-carnitine, etc., were lowly expressed; Caproyl L-carnitine, lactoyl-Leucine, etc., were highly expressed in A-MT, while mannose-6-phosphate, glycodeoxycholic acid, etc., were lowly expressed; glucose 6-phosphate, succinic acid, etc., were highly expressed in M-MT, while glycodeoxycholic acid, taurodeoxycholic acid, etc., were lowly expressed. To analyze the correlations among the differential metabolites between L-MT and L-WT, A-MT and A-WT, and M-MT and M-WT, correlation heatmaps were made for the differential metabolites (Figure 2F–H). The results showed that among the differential metabolites in L-MT and L-WT, propionic acid and succinic acid, etc., were significantly positively correlated, while 2-piperidone and 2-hydroxyglutarate, etc., were significantly negatively correlated; among the differential metabolites in A-MT and A-WT, 3a,7b,12a-trihydroxy-5-oxo-cholanoyl glycine and taurodeoxycholic acid, etc., were significantly positively correlated, while hexanoyl-L-carnitine and 4-guanidinobutyric acid, etc., were significantly negatively correlated; among the differential metabolites in M-MT and M-WT, glycodeoxycholic acid and 3a,7b,12a-trihydroxy-5-oxo-cholanoyl glycine, etc., were significantly positively correlated, while 4-guanidinobutyric acid and methyl ecdysterone, etc., were significantly negatively correlated.

**Figure 3:**
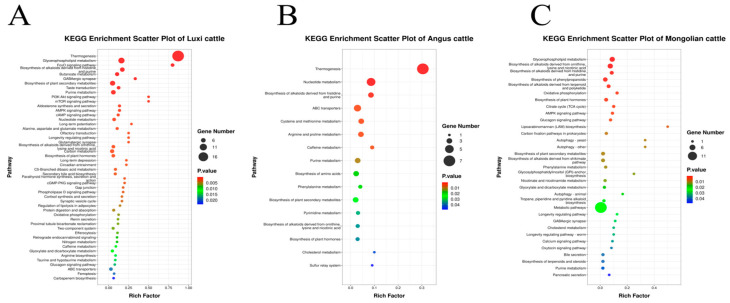
Differential metabolite-rich KEGG pathways. (**A**–**C**) bubble plots of differential metabolic pathways between L-MT and L-WT, A-MT and A-WT, and M-WT and M-MT.

To explore the metabolic pathways in which the differential metabolites between L-MT, A-MT, and M-MT and L-WT, A-WT, and M-WT are involved, KEGG pathway enrichment analysis was performed. The results are shown in Figure 3. A total of 57 metabolic pathways were enriched in the differential metabolites of Luxi cattle, including glycerophospholipid metabolism, purine metabolism, taste transduction, and others. For Angus cattle, 16 metabolic pathways were enriched in the significantly differential metabolites, such as nucleotide metabolism, alkaloid synthesis, amino acid metabolism, and so on. In Mongolian cattle, 33 metabolic pathways were enriched in the significantly differential metabolites, including glycerophospholipid metabolism, alkaloid synthesis, oxidative phosphorylation, and others. The enriched metabolic pathways were correlated with meat quality.

**Figure 4:**
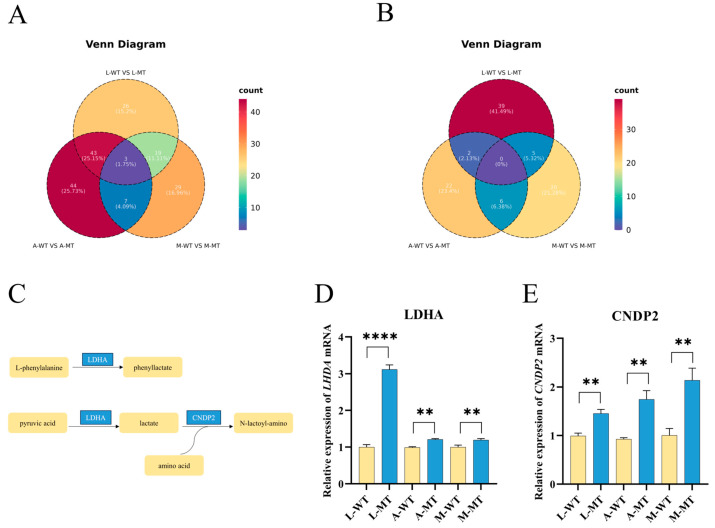
Differential metabolite analysis. (**A**) Venn diagram of upregulated differential metabolites between L-MT and L-WT, A-MT and A-WT, and M-WT and M-MT; (**B**) Venn diagram of downregulated differential metabolites between L-MT and L-WT, A-MT and A-WT, and M-WT and M-MT; (**C**) metabolic pathways related to the three common upregulated differential metabolites; (**D**,**E**) RT-qPCR experimental results. ** indicating *p* < 0.01, **** indicating *p* < 0.0001.

To explore the differential metabolites between the three breeds of MSTN gene-edited cattle and non-gene-edited cattle, Venn analysis was carried out on the upregulated and downregulated differential metabolites between L-MT and L-WT, A-MT and A-WT, and M-MT and M-WT. It was discovered that there were three significantly upregulated differential metabolites in common (Figure 4A), namely lactoyl-valine, 3-phenyllactic acid, and lactoyl-methionine, while no common downregulated differential metabolites were found (Figure 4B).

Through the analysis of three common differential metabolites, we found that the metabolic pathways of these metabolites are closely related to the LDHA and CNDP2 genes. To further investigate the expression of these two genes in MSTN gene-edited cattle, we employed quantitative real-time polymerase chain reaction (RT-qPCR) to quantitatively measure the expression of LDHA and CNDP2 genes. In the experiment, we first extracted total RNA from tissue samples of MSTN gene-edited cattle and unedited cattle, then reverse-transcribed the RNA into cDNA. Specific primers were used to amplify and quantify the LDHA and CNDP2 genes. By calculating the relative expression levels of each gene, we obtained data on the gene expression differences between the two cattle groups. The results showed that the expression levels of LDHA and CNDP2 genes in MSTN gene-edited cattle were significantly higher than in unedited cattle (Figure 4D,E and Table S4). This result is consistent with the changes in differential metabolites observed in the metabolomics analysis. The metabolomics analysis revealed that the metabolic pathways of these differential metabolites are closely associated with the LDHA and CNDP2 genes, while the RT-qPCR results suggest that MSTN gene editing may affect these two genes’ expression, thereby influencing related metabolic pathways and causing changes in metabolites. This finding provides new insights into understanding the role of MSTN gene editing in animal metabolic regulation and lays the foundation for further research into its potential biological mechanisms. In conclusion, the RT-qPCR results not only validate the conclusions drawn from the metabolomics analysis but also provide experimental evidence for the role of the LDHA and CNDP2 genes in MSTN gene-edited cattle, further illustrating the profound impact of MSTN gene editing on metabolic regulation in cattle.

## 4. Discussion

### 4.1. Meat Quality Traits of MSTN Gene-Edited Cattle

In this study, the meat quality traits of three breeds of MSTN gene-edited cattle and non-gene-edited cattle were measured. It was observed that the meat pH, shear force, and intramuscular fat content of MSTN gene-edited cattle were significantly reduced compared to those of non-gene-edited cattle (*p* < 0.05). The pH value of meat can reflect the freshness of the meat, and the reasonable pH range of beef is 5.4–5.8 [19]. Some studies have indicated that the taste of high pH meat is poorer [20]. Related research has found that the meat tenderness of MSTN gene-edited cattle is relatively high, possibly due to the lower collagen content in the muscle and the loose and thin collagen capsule structure [21,22]. In the study by Li Xin et al., it was discovered that the MSTN gene might increase the tenderness of beef from the aspect of muscle fibers [23], which is consistent with the finding in this study that the shear force of MSTN gene-edited cattle is lower and the tenderness is better compared to non-gene-edited cattle. Regarding nutritional components, the protein content, intramuscular fat content, and moisture content of both MSTN gene-edited cattle and non-gene-edited cattle are within the normal range. The intramuscular fat content of MSTN gene-edited cattle is significantly lower than that of non-gene-edited cattle (*p* < 0.05), which is the same as the research results of Zhu Lin et al. [24]. Generally, consumers’ demand for healthy beef is low fat and high protein, and the significantly lower intramuscular fat content of MSTN gene-edited cattle in this study is in line with people’s pursuit of healthy beef. The decrease in pH value, increase in tenderness, and decrease in fat content in MSTN gene-edited cattle have all contributed to the improvement of meat quality traits. There was no significant difference in the water-holding capacity of meat between MSTN gene-edited cattle and non-gene-edited cattle. In the study by Bojiang Li et al. [25], MSTN was identified as a key candidate gene responsible for drip loss. However, our results showed no significant differences in cooking loss and drip loss values between MSTN gene-edited cattle and non-edited controls. Whether MSTN gene editing affects the water-holding capacity in MSTN-edited cattle requires further investigation and validation. In summary, compared to non-gene-edited cattle, MSTN gene-edited cattle exhibited improvements in meat quality traits.

### 4.2. Metabolomics Analysis of MSTN Gene-Edited Cattle

In this study, the metabolomic characteristics of MSTN gene-edited cattle were explored through non-targeted metabolomic analysis of the longissimus thoracis muscle tissues of MSTN gene-edited cattle and non-gene-edited cattle of three breeds. Based on the PLS-DA score plot, significant differences were found between MSTN gene-edited cattle and non-gene-edited cattle. Among them, 125 differential metabolites were discovered between L-MT and L-WT, 127 differential metabolites between A-MT and A-WT, and 89 differential metabolites between M-MT and M-WT. There were three common upregulated differential metabolites among the three breeds of MSTN gene-edited cattle and non-gene-edited cattle, namely lactoyl-valine, 3-phenyllactic acid, and lactoyl-methionine. 3-Phenyllactic acid is a chiral aromatic compound involved in phenylalanine metabolism, produced from phenylpyruvic acid through the action of lactate dehydrogenase (LDHA). It is a natural broad-spectrum antibacterial organic acid and has a good inhibitory effect on various foodborne pathogenic bacteria such as Staphylococcus aureus, Escherichia coli, and Salmonella [26]. Studies have found that phenyllactic acid reduces lipid accumulation and intracellular triglyceride levels and inhibits obesity-related inflammation in human mesenchymal stem cells [27]. The increase in phenyllactic acid can extend the meat preservation of MSTN gene-edited cattle and have a positive impact on human health. Both lactoyl-valine and lactoyl-methionine are lactoyl derivatives of amino acids, produced by lactate and amino acids through cytoplasmic nonspecific dipeptidase (CNDP2). The significant increase in lactoyl derivatives in the preparation of fermented foods serves as an indicator for assessing food maturity [28,29]. Currently, only a few articles have revealed the distinct aroma and taste of lactoyl amino acids. Hofmann et al. revealed the intrinsic taste of six lactoyl amino acids and found that it helps increase the fullness, volume, and complexity of the food matrix [30]. Also, studies have found that enzymatically synthesized lactoyl phenylalanine can be used as a flavor enhancer [31]. Current research suggests that the introduction of the lactoyl group is an effective method for regulating the gustatory activity of substances [32], and lactoyl amino acid derivatives have become compounds closely related to umami perception [33]. Moreover, RT-qPCR was performed for validation, and it was found that the gene expression levels of three enzymes related to differential metabolites were elevated in MSTN gene-edited cattle. In this study, it was found that the upregulation of lactoyl amino acid metabolites in MSTN gene-edited cattle may enhance the flavor of MSTN gene-edited cattle.

## 5. Conclusions

In this study, it was found that compared with non-gene-edited cattle, MSTN gene-edited cattle had significantly lower meat pH, shear force, and intramuscular fat content, while no significant differences were observed in water-holding capacity. Overall, the meat quality traits of MSTN gene-edited cattle were improved. Further, combined with metabolomics analysis there were three upregulated differential metabolites shared between the three breeds of MSTN gene-edited and non-gene-edited cattle, namely lactoyl-valine, 3-phenyllactic acid, and lactoyl-methionine, which were found to enhance meat flavor in related studies. To sum up, the meat quality of MSTN gene-edited cattle has been improved compared to that of non-gene-edited cattle.

## Figures and Tables

**Table 1:** Specific primer sequences.

Gene Name	Forward Primer	Reverse Primer	Product Lengths
LDHA	GCTGGATGGCACTTACCTTGT	TGGACTAGGCACCTTGGCTAA	168
CNDP2	AGGTGGTCGGCAAGTTCTC	CAGGTAGTGAGGGTGGTTGAA	182
GAPDH	CAAGTTCAACGGCACAGTCAAG	CACATACTCAGCACCAGCATCA	124

**Table 2:** L-MT, A-MT, M-MT, L-WT, A-WT, M-WT Meaty traits (X ± SD).

Items	Muscle	L-WT	L-MT	A-WT	A-MT	M-WT	M-MT
**pH**	upper brain	5.72 ± 0.14 ^a^	5.44 ± 0.04 ^b^	5.69 ± 0.05 ^a^	5.47 ± 0.12 ^b^	5.73 ± 0.05 ^a^	5.47 ± 0.08 ^b^
	eye meat	5.74 ± 0.07 ^a^	5.44 ± 0.04 ^c^	5.68 ± 0.07 ^ab^	5.41 ± 0.04 ^c^	5.62 ± 0.10 ^b^	5.44 ± 0.03 ^c^
	external spine	5.64 ± 0.06 ^a^	5.43 ± 0.05 ^c^	5.57 ± 0.11 ^ab^	5.45 ± 0.03 ^bc^	5.68 ± 0.11 ^a^	5.46 ± 0.04 ^bc^
	internal spine	5.70 ± 0.05 ^a^	5.49 ± 0.03 ^b^	5.81 ± 0.17 ^a^	5.50 ± 0.01 ^b^	5.76 ± 0.12 ^a^	5.49 ± 0.03 ^b^
	anterior tendon	5.81 ± 0.04 ^a^	5.67 ± 0.15 ^abc^	5.72 ± 0.05 ^ab^	5.52 ± 0.07 ^bc^	5.76 ± 0.12 ^ab^	5.56 ± 0.07 ^bc^
	posterior tendon	5.76 ± 0.12 ^a^	5.56 ± 0.08 ^b^	5.69 ± 0.12 ^ab^	5.54 ± 0.08 ^b^	5.71 ± 0.03 ^ab^	5.56 ± 0.10 ^ab^
	semitendinosus	5.68 ± 0.14 ^ab^	5.50 ± 0.05 ^b^	5.57 ± 0.07 ^b^	5.50 ± 0.08 ^b^	5.79 ± 0.15 ^a^	5.52 ± 0.03 ^b^
**Shear force (N)**	upper brain	10.64 ± 0.16 ^ab^	8.98 ± 0.68 ^d^	10.02 ± 0.48 ^bcd^	9.31 ± 0.65 ^cd^	11.56 ± 0.97 ^a^	10.26 ± 0.42 ^bc^
	eye meat	11.83 ± 0.50 ^b^	10.45 ± 0.41 ^cd^	11.61 ± 0.76 ^bc^	10.06 ± 0.76 ^d^	13.17 ± 1.24 ^a^	10.68 ± 0.20 ^bcd^
	external spine	10.56 ± 0.84 ^ab^	9.55 ± 0.27 ^c^	9.30 ± 0.67 ^c^	8.31 ± 0.50 ^d^	10.74 ± 0.40 ^a^	9.81 ± 0.10 ^bc^
	internal spine	10.13 ± 0.72 ^bc^	8.51 ± 0.35 ^d^	10.44 ± 0.71 ^bc^	9.50 ± 0.15 ^cd^	12.82 ± 1.32 ^a^	10.92 ± 0.46 ^b^
	anterior tendon	22.16 ± 2.22 ^ab^	18.24 ± 0.98 ^b^	22.14 ± 2.21 ^ab^	19.68 ± 0.96 ^ab^	22.96 ± 2.75 ^a^	20.45 ± 2.49 ^ab^
	posterior tendon	22.05 ± 1.43 ^a^	19.71 ± 1.07 ^c^	24.97 ± 0.64 ^a^	23.39 ± 1.23 ^ab^	25.28 ± 0.23 ^a^	23.24 ± 2.06 ^ab^
	semitendinosus	17.56 ± 0.92 ^ab^	15.68 ± 1.50 ^bc^	16.86 ± 0.71 ^abc^	14.74 ± 0.59 ^d^	18.33 ± 0.15 ^a^	16.67 ± 0.62 ^bc^
**Extrusion loss rate (%)**	upper brain	4.30 ± 0.16 ^c^	4.76 ± 0.20 ^c^	4.42 ± 0.24 ^b^	4.56 ± 0.01 ^ab^	4.91 ± 0.23 ^a^	4.65 ± 0.12 ^ab^
	eye meat	5.47 ± 0.31 ^a^	5.63 ± 0.17 ^a^	5.77 ± 0.35 ^a^	5.83 ± 0.72 ^a^	5.33 ± 0.19 ^a^	5.55 ± 0.39 ^a^
	external spine	5.15 ± 0.09 ^a^	5.28 ± 0.08 ^a^	5.25 ± 0.42 ^a^	4.82 ± 0.68 ^a^	5.05 ± 0.58 ^a^	5.64 ± 0.25 ^a^
	internal spine	5.19 ± 1.01 ^a^	5.30 ± 0.83 ^a^	4.43 ± 0.47 ^a^	5.36 ± 0.73 ^a^	4.66 ± 0.97 ^a^	4.82 ± 0.40 ^a^
	anterior tendon	1.96 ± 0.05 ^bc^	1.72 ± 0.16 ^c^	3.25 ± 0.71 ^a^	3.24 ± 0.73 ^a^	2.77 ± 0.30 ^ab^	3.24 ± 0.36 ^a^
	posterior tendon	2.08 ± 0.33 ^b^	2.59 ± 0.12 ^b^	3.48 ± 0.31 ^a^	3.57 ± 0.40 ^a^	2.27 ± 0.41 ^b^	2.62 ± 0.33 ^b^
	semitendinosus	5.23 ± 1.19 ^bc^	4.24 ± 1.15 ^c^	4.78 ± 0.30 ^bc^	4.58 ± 0.36 ^c^	6.14 ± 0.35 ^ab^	6.93 ± 0.50 ^a^
**Cooking loss rate (%)**	upper brain	31.42 ± 2.19 ^ab^	29.43 ± 1.40 ^b^	33.16 ± 0.76 ^a^	32.70 ± 2.03 ^a^	32.53 ± 1.25 ^a^	33.56 ± 0.90 ^a^
	eye meat	23.06 ± 0.32 ^c^	24.47 ± 1.15 ^c^	32.27 ± 1.56 ^b^	31.83 ± 1.46 ^b^	34.40 ± 1.08 ^a^	34.56 ± 0.91 ^a^
	external spine	30.57 ± 2.00 ^abc^	29.70 ± 0.67 ^bc^	31.76 ± 2.94 ^abc^	28.40 ± 1.20 ^c^	33.73 ± 1.16 ^a^	32.00 ± 1.85 ^ab^
	internal spine	32.82 ± 2.55 ^ab^	33.85 ± 1.02 ^ab^	30.77 ± 1.46 ^ab^	32.19 ± 2.02 ^b^	34.00 ± 1.73 ^ab^	35.63 ± 1.03 ^a^
	anterior tendon	33.99 ± 2.66 ^a^	34.00 ± 0.61 ^a^	29.40 ± 3.40 ^b^	31.27 ± 1.61 ^ab^	29.27 ± 0.71 ^b^	29.26 ± 0.68 ^b^
	posterior tendon	31.03 ± 1.56 ^a^	30.43 ± 2.50 ^a^	31.20 ± 0.61 ^a^	31.50 ± 1.93 ^a^	32.53 ± 0.85 ^a^	32.56 ± 1.16 ^a^
	semitendinosus	31.17 ± 2.92 ^ab^	31.67 ± 1.05 ^a^	28.40 ± 0.92 ^b^	28.44 ± 0.75 ^b^	31.67 ± 1.07 ^a^	31.43 ± 1.22 ^a^
**Moisture (%)**	upper brain	69.90 ± 0.17 ^a^	69.83 ± 0.64 ^a^	69.90 ± 0.10 ^a^	69.73 ± 0.21 ^a^	69.60 ± 0.20 ^a^	69.30 ± 0.20 ^a^
	eye meat	70.07 ± 0.06 ^ab^	70.37 ± 0.23 ^a^	70.03 ± 0.15 ^b^	70.00 ± 0.10 ^b^	70.00 ± 0.10 ^b^	70.30 ± 0.20 ^ab^
	external spine	69.86 ± 0.12 ^b^	70.10 ± 0.17 ^ab^	70.30 ± 0.20 ^a^	70.03 ± 0.06 ^ab^	70.23 ± 0.25 ^a^	70.13 ± 0.06 ^ab^
	internal spine	70.20 ± 0.46 ^a^	70.43 ± 0.29 ^a^	69.90 ± 0.30 ^a^	70.10 ± 0.20 ^a^	69.90 ± 0.10 ^a^	70.13 ± 0.06 ^a^
	anterior tendon	70.37 ± 0.06 ^a^	70.43 ± 0.12 ^a^	70.23 ± 0.06 ^ab^	70.13 ± 0.15 ^b^	70.43 ± 0.15 ^a^	70.23 ± 0.06 ^ab^
	posterior tendon	70.33 ± 0.47 ^a^	70.46 ± 0.58 ^a^	70.00 ± 0.10 ^a^	70.00 ± 0.10 ^a^	69.93 ± 0.25 ^a^	70.20 ± 0.10 ^a^
	semitendinosus	70.16 ± 0.29 ^a^	70.17 ± 0.12 ^a^	70.30 ± 0.20 ^a^	70.10 ± 0.00 ^a^	70.50 ± 0.10 ^a^	70.30 ± 0.20 ^a^
**Protein (%)**	upper brain	20.30 ± 0.00 ^a^	20.30 ± 0.17 ^a^	20.07 ± 0.06 ^a^	20.13 ± 0.15 ^a^	20.13 ± 0.05 ^a^	20.30 ± 0.20 ^a^
	eye meat	20.30 ± 0.00 ^a^	20.47 ± 0.06 ^a^	20.10 ± 0.10 ^a^	20.10 ± 0.06 ^a^	20.03 ± 0.06 ^a^	20.13 ± 0.06 ^a^
	external spine	20.36 ± 0.06 ^ab^	20.30 ± 0.00 ^bc^	19.97 ± 0.15 ^abc^	20.00 ± 0.00 ^c^	20.13 ± 0.06 ^abc^	20.20 ± 0.10 ^a^
	internal spine	20.26 ± 0.12 ^ab^	20.47 ± 0.15 ^a^	20.13 ± 0.15 ^bc^	20.03 ± 0.06 ^c^	20.20 ± 0.00 ^bc^	20.20 ± 0.10 ^bc^
	anterior tendon	20.23 ± 0.12 ^a^	20.30 ± 0.20 ^a^	20.07 ± 0.15 ^a^	20.06 ± 0.06 ^a^	20.13 ± 0.06 ^a^	20.20 ± 0.10 ^a^
	posterior tendon	20.23 ± 0.12 ^ab^	20.33 ± 0.06 ^a^	20.10 ± 0.10 ^ab^	20.10 ± 0.17 ^b^	20.13 ± 0.06 ^ab^	20.03 ± 0.06 ^b^
	semitendinosus	20.43 ± 0.12 ^a^	20.30 ± 0.00 ^ab^	20.20 ± 0.10 ^bc^	20.10 ± 0.00 ^c^	20.23 ± 0.06 ^bc^	20.13 ± 0.15 ^bc^
**Intramuscular Fat (%)**	upper brain	12.46 ± 0.40 ^a^	10.30 ± 0.17 ^c^	10.26 ± 0.06 ^c^	9.66 ± 0.11 ^d^	11.40 ± 0.10 ^b^	10.23 ± 0.15 ^c^
	eye meat	11.43 ± 0.40 ^a^	10.00 ± 0.35 ^bc^	10.47 ± 0.40 ^b^	9.17 ± 0.12 ^d^	11.23 ± 0.66 ^a^	9.70 ± 0.10 ^cd^
	external spine	12.73 ± 0.81 ^a^	11.36 ± 0.75 ^b^	11.37 ± 0.32 ^c^	10.33 ± 0.72 ^c^	10.13 ± 0.06 ^c^	10.50 ± 0.00 ^bc^
	internal spine	11.27 ± 0.06 ^a^	8.83 ± 0.06 ^c^	10.90 ± 0.61 ^ab^	8.90 ± 0.80 ^c^	10.23 ± 0.06 ^b^	8.50 ± 0.40 ^c^
	anterior tendon	9.87 ± 0.28 ^abc^	9.23 ± 0.11 ^c^	10.07 ± 0.75 ^ab^	9.20 ± 0.10 ^c^	10.33 ± 0.46 ^a^	9.55 ± 0.05 ^bc^
	posterior tendon	10.07 ± 0.12 ^cd^	9.57 ± 0.12 ^d^	10.13 ± 0.12 ^bc^	9.73 ± 0.25 ^c^	10.83 ± 0.65 ^a^	10.63 ± 0.15 ^ab^
	semitendinosus	11.03 ± 0.40 ^ab^	10.13 ± 0.58 ^c^	9.50 ± 0.87 ^cd^	9.13 ± 0.15 ^d^	11.43 ± 0.06 ^a^	10.30 ± 0.20 ^bc^

^a–d^ Mean values with different superscripts within the same row indicate significant differences (*p* < 0.05).

## Data Availability

The original contributions presented in the study are included in the article/Supplementary Materials, further inquiries can be directed to the corresponding author.

## Supplementary Materials

The following supporting information can be downloaded at: https://www.mdpi.com/article/10.3390/ani15010047/s1, Table S1: Top 30 differential metabolites between L-WT and L-MT; Table S2: Top 30 differential metabolites between A-WT and A-MT; Table S3: Top 30 differential metabolites between M-WT and M-MT; Table S4: RT-qPCR quantitative results.
